# Prediction of Pelvic Organ Prolapse Postsurgical Outcome Using Biomaterial-Induced Blood Cytokine Levels: Machine Learning Approach

**DOI:** 10.2196/40402

**Published:** 2023-05-31

**Authors:** Mihyun Lim Waugh, Nicholas Boltin, Lauren Wolf, Jane Goodwin, Patti Parker, Ronnie Horner, Matthew Hermes, Thomas Wheeler, Richard Goodwin, Melissa Moss

**Affiliations:** 1 Biomedical Engineering Program, University of South Carolina Columbia, SC United States; 2 Biomedical Sciences, University of South Carolina School of Medicine - Greenville Greenville, SC United States; 3 Prisma Health Greenville Greenville, SC United States; 4 Department of Health Services Research and Administration, University of Nebraska Medical Center Omaha, NE United States; 5 Department of Bioengineering, Clemson University Clemson, SC United States; 6 Spartanburg Regional Healthcare System Spartanburg, SC United States; 7 Department of Chemical Engineering, University of South Carolina Columbia, SC United States

**Keywords:** pelvic organ prolapse, polypropylene mesh, inflammatory response, cytokines, principal component analysis, supervised machine learning models, surgical outcome prediction, biomaterial, repair surgery

## Abstract

**Background:**

Pelvic organ prolapse (POP) refers to symptomatic descent of the vaginal wall. To reduce surgical failure rates, surgical correction can be augmented with the insertion of polypropylene mesh. This benefit is offset by the risk of mesh complication, predominantly mesh exposure through the vaginal wall. If mesh placement is under consideration as part of prolapse repair, patient selection and counseling would benefit from the prediction of mesh exposure; yet, no such reliable preoperative method currently exists. Past studies indicate that inflammation and associated cytokine release is correlated with mesh complication. While some degree of mesh-induced cytokine response accompanies implantation, excessive or persistent cytokine responses may elicit inflammation and implant rejection.

**Objective:**

Here, we explore the levels of biomaterial-induced blood cytokines from patients who have undergone POP repair surgery to (1) identify correlations among cytokine expression and (2) predict postsurgical mesh exposure through the vaginal wall.

**Methods:**

Blood samples from 20 female patients who previously underwent surgical intervention with transvaginal placement of polypropylene mesh to correct POP were collected for the study. These included 10 who experienced postsurgical mesh exposure through the vaginal wall and 10 who did not. Blood samples incubated with inflammatory agent lipopolysaccharide, with sterile polypropylene mesh, or alone were analyzed for plasma levels of 13 proinflammatory and anti-inflammatory cytokines using multiplex assay. Data were analyzed by principal component analysis (PCA) to uncover associations among cytokines and identify cytokine patterns that correlate with postsurgical mesh exposure through the vaginal wall. Supervised machine learning models were created to predict the presence or absence of mesh exposure and probe the number of cytokine measurements required for effective predictions.

**Results:**

PCA revealed that proinflammatory cytokines interferon gamma, interleukin 12p70, and interleukin 2 are the largest contributors to the variance explained in PC 1, while anti-inflammatory cytokines interleukins 10, 4, and 6 are the largest contributors to the variance explained in PC 2. Additionally, PCA distinguished cytokine correlations that implicate prospective therapies to improve postsurgical outcomes. Among machine learning models trained with all 13 cytokines, the artificial neural network, the highest performing model, predicted POP surgical outcomes with 83% (15/18) accuracy; the same model predicted POP surgical outcomes with 78% (14/18) accuracy when trained with just 7 cytokines, demonstrating retention of predictive capability using a smaller cytokine group.

**Conclusions:**

This preliminary study, incorporating a sample size of just 20 participants, identified correlations among cytokines and demonstrated the potential of this novel approach to predict mesh exposure through the vaginal wall following transvaginal POP repair surgery. Further study with a larger sample size will be pursued to confirm these results. If corroborated, this method could provide a personalized medicine approach to assist surgeons in their recommendation of POP repair surgeries with minimal potential for adverse outcomes.

## Introduction

Pelvic organ prolapse (POP), defined as symptomatic descent of the vagina and surrounding pelvic organs, affects approximately 50% of parous women and 6% of nonparous women between ages 20 and 59 years [1], with almost 300,000 POP surgeries performed per year [2]. To reduce anatomical recurrence, surgical treatment may include the insertion of polypropylene mesh into the vaginal wall to provide mechanical support and reinforcement of the prolapsed organs. Unfortunately, postsurgical mesh complication, predominantly mesh exposure through the vaginal wall, occurs with some frequency and results in decreased quality of life, leaving patients with costly residual symptoms and emotional distress [3]. Patients may elect for surgical reintervention to revise or remove the mesh implantation. In fact, according to Reid et al [4], 37 (8%) out of 482 patients underwent further surgery to remove the mesh, and 7 (2%) patients repeated the prolapse surgery. These complications provoked the removal of transvaginal mesh kits from the market by the Food and Drug Administration in 2019. A clinical decision support tool to better inform both patients and surgeons about the risk of complications following POP surgery may allow for the reintroduction of this advantageous surgical augmentation.

Inflammatory responses are associated with mesh exposure due to asymptomatic mesh infection that inhibits the mesh from integrating with the surrounding environment [5]. While some degree of mesh-induced cytokine response is necessary for successful implantation, excess or unattenuated cytokine response could result in chronic inflammation and implant rejection. As chronic inflammation progresses, granulation tissues formed during the foreign body reaction will evolve into mesh encapsulation by regular dense connective tissue and myofibroblast-induced contracture around the implant, which can result in mesh exposure [6,7]. The balance between proinflammatory and anti-inflammatory agents is critical in achieving successful mesh implantation, and this balance may be influenced by the individual's response to the implant material. Thus, leveraging a patient’s immune response to the biomaterial could facilitate the prediction of postsurgical outcomes.

Leveraging a patient-specific, multifaceted immune response for the prediction of postsurgical complications is an ideal problem for the application of principal component analysis (PCA) and supervised machine learning models. In fact, this approach has been used to predict complications following other surgical procedures as well as progressive disease outcomes. In a liver transplant study, Raji and Vinod Chandra [8] applied PCA to a composite medical data set comprised of donors’ medical information as well as the recipients’ medical history and implemented an artificial neural network to predict the long-term survival of liver transplant patients. In an oral cancer retrospective study by Chu et al [9], PCA along with bivariate analyses were used to highlight correlated variables from the patient data, which included patient demographics and clinicopathological tumor data (including tumor sites, disease staging, etc), and to predict oral cancer progress.

PCA and supervised machine learning have also been applied to biological measurements for predicting medical outcomes. Tseng et al [10] built a predictive model for cardiac surgery–associated acute kidney injury (AKI) using preoperative biochemistry data in combination with patient demographic characteristics and clinical condition. Incorporating a different type of biological measurement, a glioblastoma study by Akbari et al [11] used PCA and support vector machines to distinguish multiparametric magnetic resonance imaging signatures and quantify the patterns to predict regions of tumor recurrence after surgery. Chen et al [12] demonstrated that specifically including immune data in predictive models enhances predictive capacity. These researchers implemented machine learning models using individual patient immune data, such as blood cytokine levels, to predict severe AKI after cardiac surgery and found that this approach provided a far superior prediction tool compared to a clinical factor–based model [12].

The application of PCA and machine learning to predict postsurgical complications in women after POP surgery has also shown promising results. In the study of Jelovsek et al [13], statistical modeling uses 32 candidate risk factors (ie, age, race, smoking history, etc) identified by consensus with surgical outcomes to predict postsurgical complications. This approach of using personalized preoperative decision-making based on the individual’s medical history presents a better predictive model to postsurgical complications and offers a more effective decision support tool than the practice of counseling patients using average success rates reported from large, randomized studies [13]. However, this predictive method does not leverage the patient’s potential immune response to the surgery involving polypropylene mesh.

In this preliminary study, we explored the levels of baseline and stimulus-induced cytokines in blood isolated from patients who had undergone POP repair surgery with a polypropylene mesh. Proinflammatory and anti-inflammatory cytokine levels from these data were analyzed using PCA to establish the principal components (PCs) and to identify associative or opposing trends among cytokines. In addition, supervised machine learning models were applied to demonstrate predictive capabilities when models were trained with either all 13 cytokines or a smaller group of 7 cytokines determined most effective by a random forest method. The results demonstrate that leveraging PCA and supervised machine learning models to predict outcomes of vaginal mesh implantation has the potential to benefit future patients when they are faced with this surgical decision, which carries a relatively high risk of unsuccessful surgical outcome.

## Methods

### Study Population

In total, 20 healthy, nonpregnant female participants aged 56-89 years at Prisma Health Greenville Memorial Hospital with a history of surgical intervention to correct POP via a procedure that used polypropylene mesh were selected for the study. The participants, who were not matched, included 10 who experienced postsurgical mesh complication in which the implanted mesh protruded through the vaginal wall (also referred to as mesh exposure) and 10 participants who did not experience this complication post surgery. This sample size was estimated as an effective cohort for the pilot study using a 1-tailed *t* test based on an a priori power analysis, which indicates the number of patients for a given theoretical minimum study power as a function of the expected difference between patients with and those without mesh exposure, or the Cohen *d*. We assumed a conservative study power of 0.80 and a 100%, or 2-fold, difference in the level of a given cytokine between individuals with and without mesh exposure, equivalent to a Cohen *d* of 1. Here, 20 patients with equal distribution among the 2 groups are needed to observe the difference with a probability of .1. Participants with POP recurrence or taking medications that would alter inflammatory response were excluded from this study.

### Ethics Approval

The study protocol was approved by the institutional review board (IRB) of Prisma Health (Pro00067964). Informed consent from all study participants was obtained using an IRB-approved informed consent form. All samples collected and data analyzed were deidentified and followed IRB protocol.

### Blood Sample Collection and Processing

Blood samples were obtained from the 20 selected participants. Approximately 12 mL of blood was drawn from the upper extremity of each participant into 3 BD Vacutainer EDTA-coated tubes. Deidentified blood samples were then transferred on ice to a laboratory facility at the University of South Carolina School of Medicine Greenville for immediate processing. Each participant’s blood sample was divided into equal aliquots for 24-hour incubation at 37 °C under 3 distinct conditions: (1) incubation with inflammatory agent lipopolysaccharide (LPS) at 20 ng/mL (positive control), (2) incubation with sterile polypropylene mesh area of 2 cm × 2 cm (experimental), and (3) incubation alone (negative control). After incubation, the plasma layer was collected following centrifugation (1500 × g, 10 min, 4 °C) and immediately stored at –80 °C.

### Measurement of Blood Cytokine Levels

Cytokine levels in each blood sample were quantified using the bead-based MILLIPLEX Human Cytokine/Chemokine/Growth Factor Panel A—Immunology Multiplex Assay (EMD Millipore Corp), which is composed of analytes for target cytokines interleukin 1α (IL-1α), IL-1β, IL‑2, IL-4, IL-6, IL-8, IL-10, IL-12p40, IL-12p70, IL-17A, interferon-gamma (IFN-γ, tumor necrosis factor-alpha (TNF-α), and granulocyte-macrophage colony-stimulating factor (GM-CSF). Frozen plasma samples were thawed at room temperature and analyzed following Milliplex protocol guidelines. Cytokine concentrations were measured using a Bio-Plex 200 (Bio-Rad) and Bio-Plex Manager software (Bio-Rad). Sample volume was doubled to ensure measurable levels of cytokines, and assay output data were adjusted to reflect concentrations in plasma samples. Each multiplex assay was performed in duplicate, and cytokine levels were evaluated in 3 independent measurements.

### Data Analysis

#### Overview

Cytokine data gathered from the multiplex immunoassay were analyzed using data mining and predictive analytical methods. PCA was used to identify important cytokines by studying their contributions to each PC as well as to discern associations between cytokines. Supervised machine learning models were created to determine whether cytokine levels can accurately predict which patients are more likely to experience mesh exposure post surgery.

#### Descriptive Analytics

The statistical programming language R (version 4.1.2; R Foundation) was used to analyze raw cytokine data values generated from the multiplex immunoassay. The imported data structure contained 60 observations (20 participants × 3 independent measurements) and 40 total variable fields (13 cytokines × 3 blood treatments + 1 target variable). The target variable was the participant’s outcome, which indicated a postsurgical complication that participants might have experienced following POP surgery. Observations marked “presence” represent participants who experienced mesh exposure through the vaginal wall. Observations marked “absence” represent participants who did not experience any mesh exposure through the vaginal wall. Univariate and multivariate methods were used to explore the data set, including identifying missing values, analyzing outliers, and visualizing frequency distributions.

#### PCA

PCA was performed using the FactoMineR package (version 2.4; R Foundation) [14] to identify associations between cytokines [15]. Before analysis, data transformations were performed on each variable to correct for skewness in the distribution. The amount of skewness was calculated to assess the symmetry of distribution for each variable using equation 1, where
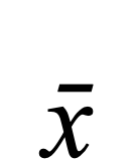
is the sample mean and *x_i_* and *n* are the individual observations and number of observations, respectively, within the sample [16]. Each cytokine’s distribution was corrected for skewness using either a natural logarithm, square root, or inverse square root method. Using equation 2, *z*-score standardization was also applied to scale each cytokine variable, thus ensuring that the mean was equal to 0 and SD equal to 1. Biplots were created to visualize PCs with the highest degree of variance explained. The eigenvectors were overlayed on the biplots to visualize correlations and identify hidden patterns between cytokines.

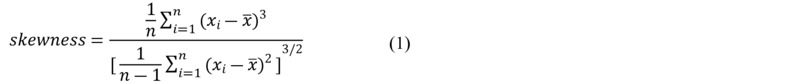

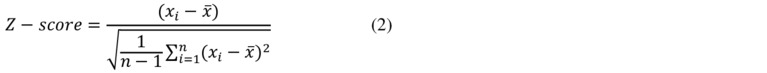





#### Predictive Analytics

Supervised machine learning models were created using the caret package (version 6.0-90; R Foundation) [17] in the R programming language. The 4 models trained were decision tree, logistic regression, Naive Bayes, and artificial neural network. This approach focused on the data set from the experimental group only (cytokine expression for blood incubated with polypropylene mesh). Prior to creating the predictive models, the original data were split using an industry standard of 70% for training and 30% for testing. Each group contained an equal distribution of participants who did or did not experience postsurgical mesh exposure through the vaginal wall—the prediction target for each model. Each model was then trained using the 70% (42/60) subset and a cross-validation training control. A 10-fold cross-validation with 25% (15/60) left out replicated 3 times was used on each model to avoid bias and overfitting. From this, training accuracies are reported. Additional testing was performed for the prediction accuracy of each model using the 30% (18/60) test data. Prediction accuracies are reported along with sensitivity and specificity for the prediction of participants to experience postsurgical mesh exposure.

Additionally, this process was replicated to study the effects of reducing the number of cytokines needed to predict a postsurgical mesh exposure. A random forest algorithm was used to select important cytokines for this study. These models were trained and tested for accuracy, sensitivity, and specificity as detailed above. The results are compared to models trained with all 13 cytokines.

## Results

### PCA

**Figure 1:**
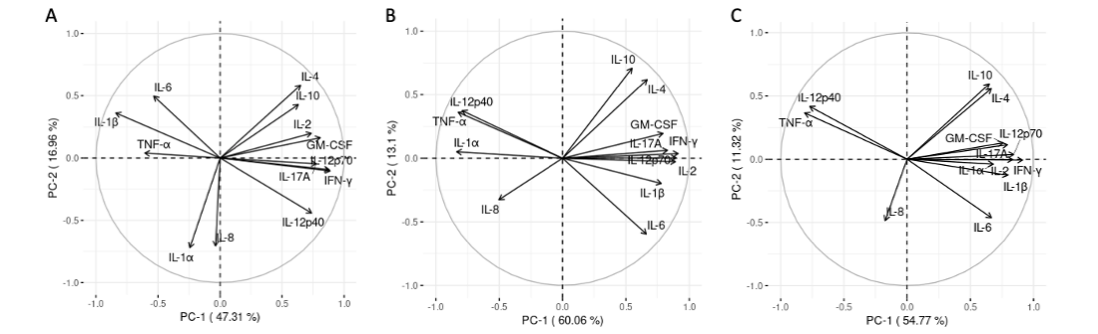
PCA was performed using cytokine levels in blood samples of postsurgical POP subjects; the analysis included 60 blood samples (20 subjects × 3 blood treatments), wherein each sample was evaluated in 3 independent measurements performed in duplicate. Biplots illustrating individual cytokines were constructed for blood samples incubated in the presence of LPS (A), incubated in the presence of polypropylene mesh (B), or incubated alone (C). Arrow direction indicates the cytokine correlation; arrow length indicates the magnitude of the variation. IL-1α: interleukin-1 alpha; IL-1β: interleukin-1 beta; IL-2: interleukin-2; IL-4: interleukin-4; IL-6: interleukin-6; IL-8: interleukin-8; IL-10: interleukin-10; IL-12p40: interleukin-12p40; IL-12 p70: interleukin-12p70; IL-17A: interleukin-17A; IFN-γ: interferon gamma; TNF-α: tumor necrosis factor-alpha; GM-CSF: granulocyte-macrophage colony-stimulating factor; PC: principal component.

To identify significant associations among the cytokines, PCA was used to examine a total of 60 blood samples (20 participants × 3 blood treatments). Among the 20 participants, 10 experienced postsurgical mesh exposure through the vaginal wall and 10 did not. Figure 1 depicts a biplot of each blood treatment and summarizes the intercorrelated relationships among individual inflammatory mediators. The combined variances explained for PC 1 and PC 2 in blood samples incubated with LPS (Figure 1A), polypropylene mesh (Figure 1B), or alone (Figure 1C) were approximately 64%, 73%, and 66%, respectively. In all 3 treatment groups, IL-10 and IL-4 align in the same directions, as do IL-12p70 and IFN-γ, indicating a positive correlation for both of these cytokine pairs. In contrast, IL-6 and IL-12p40 were negatively correlated when comparing stimulation of blood via LPS (Figure 1A) versus polypropylene mesh (Figure 1B). Only IL-1α displayed a negative correlation when comparing blood incubated with polypropylene mesh (Figure 1B) versus blood incubated alone (Figure 1C).

**Figure 2:**
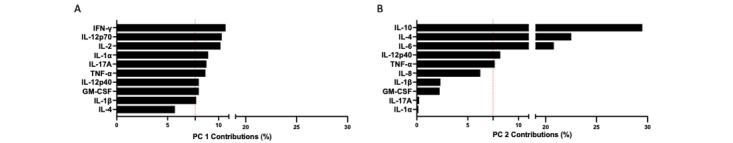
PCA was performed using cytokine levels in patient blood samples incubated with polypropylene mesh; the analysis included 20 blood samples (20 subjects × 1 blood treatment), wherein each sample was evaluated in 3 independent measurements performed in duplicate. Each cytokine’s contribution to PC 1 (A) and PC 2 (B) was determined. The dashed line at 7% corresponds to the expected value if the contribution were uniform. IL-1α: interleukin-1 alpha; IL-1β: interleukin-1 beta; IL-2: interleukin-2; IL-4: interleukin-4; IL-6: interleukin-6; IL-8: interleukin-8; IL-10: interleukin-10; IL-12p40: interleukin-12p40; IL-12p70: interleukin-12p70; IL-17A: interleukin-17A; IFN-γ: interferon gamma; TNF-α: tumor necrosis factor-alpha; GM-CSF: granulocyte-macrophage colony-stimulating factor; PC: principal component.

When PCA was used to examine only blood samples incubated with polypropylene mesh, PC 1 and PC 2 explained 60.1% and 13.1% of the total data variance, respectively (Figure 1B). Figure 2A displays each cytokine’s contribution to PC 1 and illustrates that IFN-γ, IL-12p70, and IL-2 are the predominant contributors to the variance explained in PC 1. In addition, IL-1α, IL-17A, and TNF-α exhibited contributions above a level expected if the contributions were uniform. All other cytokines have contributions to PC 1 similar to or less than what would be expected if the contributions of all cytokines were uniform. Figure 2B illustrates that the predominant contributors to the variance explained in PC 2 are IL-10, IL-4, and IL-6. All other cytokines have contributions to PC 2 similar to or less than what would be expected if the contribution of all cytokines were uniform.

**Figure 3:**
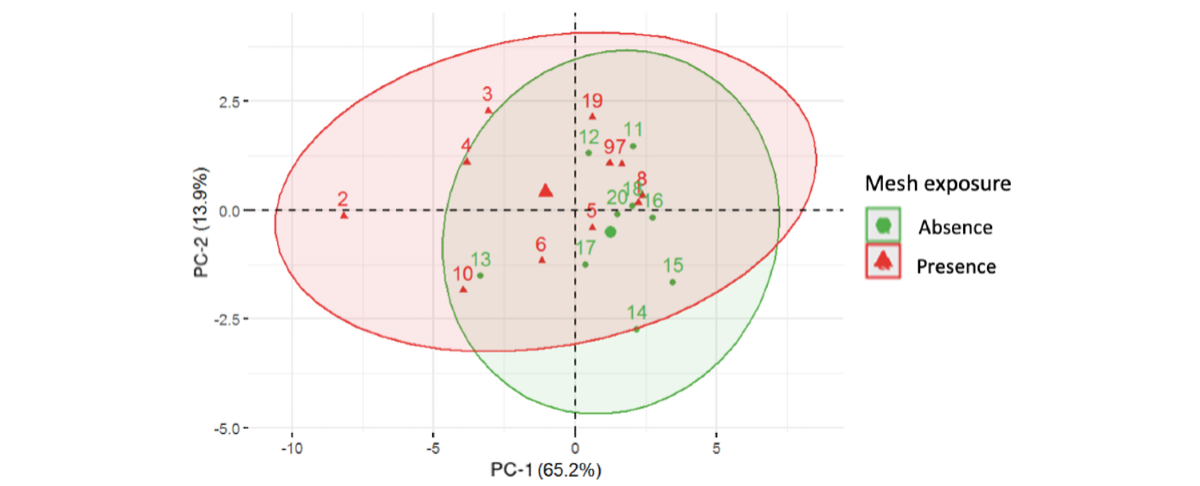
PCA was performed using cytokine levels in patient blood samples incubated with polypropylene mesh; the analysis included 20 blood samples (20 subjects × 1 blood treatment), wherein each sample is represented by the average of 3 independent measurements performed in duplicate. A biplot was constructed illustrating individual patient averages (indicated by numbers) exhibiting the presence (red triangle) or absence (green circle) of mesh exposure through the vaginal wall. Concentration ellipses draw focus to the distribution of a group with the presence (red) or absence (green) of mesh exposure. Centroids of the concentration ellipses (large symbols) indicate the mean of each group. PC: principal component.

In order to visualize associations between the participants presenting the absence or presence of postsurgical mesh exposure through the vaginal wall, a biplot illustrating individual participants was created (Figure 3). This biplot reveals a high percentage of variability represented by the first 2 PCs (79.1%). Blood samples from participants who did not experience postsurgical mesh exposure were heavily represented by positive PC 1 values, while blood samples from participants with the presence of postsurgical mesh exposure were generally represented by positive PC 2 values.

### Predictive Analysis

Four supervised machine learning models incorporating all 13 cytokines were trained using 70% (42/60) of the available 60 observations (20 participants × 3 independent measurements); the remaining 30% (18/60) was used to test the models’ accuracy when predicting the presence of mesh exposure through the vaginal wall. All 4 machine learning machines achieved at least 62% (26/42) training accuracy (Table 1). Artificial neural network achieved the highest prediction accuracy of 83% (15/18), while decision tree and Naïve Bayes both achieved a prediction accuracy of 61% (11/18). Naïve Bayes, decision tree, and artificial neural network excelled at correctly predicting patients with the presence of mesh exposure postsurgery at 89% (16/18). Artificial neural network was superior for correctly predicting patients who did not experience mesh exposure postsurgery (14/18, 78%).

**Table 1:** Summary of supervised learning model statistics. All 13 cytokines were used to predict the presence or absence of postsurgical mesh exposure through the vaginal wall; 70% (42/60) of observations were used for training, and 30% (18/60) of observations were used for testing.

Model	Training accuracy, n (%)	Prediction accuracy, n (%)	95% CI	Sensitivity, n (%)	Specificity, n (%)	Prediction, κ
Artificial neural network	33 (79)	15 (83)	0.586-0.964	14 (78)	16 (89)	0.667
Decision tree	27 (64)	11 (61)	0.57-0.827	6 (33)	16 (89)	0.222
Naïve Bayes	26 (62)	11 (61)	0.357-0.827	6 (33)	16 (89)	0.222
Logistic regression	31 (73)	9 (50)	0.260-0.740	10 (56)	8 (44)	0.000

### Predictive Analysis Using Feature Selection

**Figure 4:**
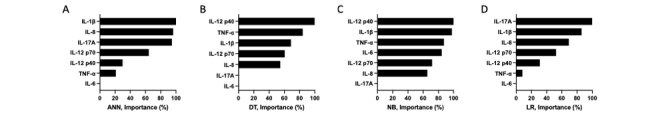
A random forest algorithm was used to identify a group of 7 cytokines capable of yielding effective predictive analysis. The importance of each cytokine is evident in individual supervised learning models: ANN (A), DT (B), NB (C), and LR (D). IL-1α: interleukin-1 alpha; IL-1β: interleukin-1 beta; IL-2: interleukin-2; IL-4: interleukin-4; IL-6: interleukin-6; IL-8: interleukin-8; IL-10: interleukin-10; IL-12p40: interleukin-12p40; IL-12p70: interleukin-12p70; IL-17A: interleukin-17A; IFN-γ: interferon gamma; TNF-α: tumor necrosis factor-alpha; GM-CSF: granulocyte-macrophage colony-stimulating factor; PC: principal component; ANN: artificial neural network; DT: decision tree; NB: Naïve Bayes; LR: logistic regression.

Additional models and predictive analyses explored whether a smaller set of cytokines could achieve similar predictive results. Feature selection using a random forest method identified a group of 7 cytokines capable of yielding effective predictive analysis: IL-1β, IL-8, IL-12p40, IL-12p70, TNF-α, IL-17A, and IL-6. Figure 4 illustrates that models exhibited variation among the importance of cytokines when implementing this more targeted group of cytokines. IL-1 and IL-8 are strongly represented in all models, while IL-6 is important in only Naïve Bayes.

Table 2 illustrates that all models achieved at least 64% (27/42) training accuracy. The logistic regression model that used the 7 selected cytokines achieved a training accuracy of 81% (34/42), a prediction accuracy of 72% (13/18), a sensitivity of 67% (12/18), and a specificity of 78% (14/18), and thus outperformed compared to the logistic regression model that incorporated all of the cytokine data. Moreover, decision tree models achieved the same result when using the selected cytokines or when all cytokines were included. The prediction accuracy in Naïve Bayes and artificial neural network models executed with the 7 selected cytokines decreased by only 5% each compared to the same models that used all of the cytokine data.

**Table 2:** Summary of supervised learning model statistics. Feature selection via random forest was used to identify a group of 7 cytokines capable of yielding effective predictive analysis. The subset of cytokines was used to predict the presence or absence of postsurgical mesh exposure through the vaginal wall; 70% (42/60) of observations were used for training, and 30% (18/60) of observations were used for testing.

Model	Training accuracy, n (%)	Prediction accuracy, n (%)	95% CI	Sensitivity, n (%)	Specificity, n (%)	Prediction, κ
Artificial neural network	34 (81)	14 (78)	0.524-0.936	12 (67)	16 (89)	0.556
Decision tree	27 (64)	11 (61)	0.356-0.827	6 (33)	16 (89)	0.222
Naïve Bayes	30 (72)	10 (56)	0.308-0.785	6 (33)	14 (78)	0.111
Logistic regression	34 (81)	13 (72)	0.465-0.903	12 (67)	14 (78)	0.444

## Discussion

### Summary

Among patients with POP who undergo mesh implantation surgery, 17% of them experience mesh exposure through the vaginal wall [18]. This rate of surgical mesh complication is significant when compared to 0.035%-5.4% mesh-related erosions reported in other mesh-based surgeries [19-23], necessitating the development of a personalized decision support tool for patients with POP. This exploratory study demonstrates a novel and efficient approach to predicting postsurgical outcomes for mesh implantation using cytokine levels in patient blood following exposure to a biomaterial. Previous studies have often used patient demographic and medical data to train machine learning programs to create predictive outcomes for POP mesh surgeries [13]. In contrast, this study uses biological material to mimic an in vivo response, thus presenting a novel, noninvasive, personalized clinical decision tool. A systematic PCA approach identifies associations among cytokines that provide physiological insight. Supervised machine learning models developed in this study demonstrate that blood cytokine measurements may be used as a predictive tool. In addition, the number of cytokine measurements needed may be reduced without compromising predictive capabilities, rendering this approach more applicable within a clinical setting.

### Principal Findings and Comparison to Prior Work

The PCA analysis illustrated in Figure 1 reveals several significant associations among the cytokines. Several cytokines display positive correlations when comparing the 2 different stimuli: LPS and polypropylene mesh. However, IL-6 and IL-12p40 are negatively correlated between these 2 treatments. Thus, these 2 cytokines may explain the different inflammatory responses induced by LPS versus polypropylene mesh. When comparing blood samples incubated alone to those incubated in the presence of polypropylene mesh, only IL-1α exhibits a negative correlation, demonstrating that this proinflammatory mediator might be specifically affected by the mesh stimulus. Furthermore, 2 pairs of cytokines positively correlate (IL-10 and IL-4; IL-12p70 and IFN-γ), indicating that one of the cytokines in each pair could be eliminated to reduce the number of cytokines tested in a clinical setting.

The cytokines that contribute most to each PC segregate into proinflammatory and anti-inflammatory cytokines (Figure 2). When cytokine data from patient blood incubated with mesh were analyzed using PCA, cytokines IFN-γ, IL-12p70, and IL-2 were the largest contributors to the variance explained in PC 1. These markers are identified as proinflammatory agents [24-26], which suggests that proinflammatory cytokines may heavily influence PC 1. In contrast, cytokines IL-10, IL-4, and IL-6 were the largest contributors to the variance explained in PC 2. IL-4 and IL-10 are prominent anti-inflammatory cytokines [25], suggesting that anti-inflammatory cytokines heavily influence PC 2. IL-6, previously thought to have proinflammatory function only, is recently recognized as potentially having both proinflammatory and anti-inflammatory roles in COVID-19 [27] and diabetes [28].

When juxtaposing the biplot of polypropylene-stimulated cytokine observations (Figure 1B) with that of mesh exposure outcome (Figure 3), it can be extrapolated that proinflammatory cytokines IL-12p40, IL-1α, and TNF-α are positioned in the region of the biplot that uniquely corresponds to surgical outcomes involving the presence of mesh exposure through the vaginal wall. Such juxtaposition suggests that IL-12p40, IL-1α, and TNF-α may be associated with the presence of postsurgical mesh exposure. These observations may inspire potential therapeutic strategies that could improve postsurgical outcome. For example, the surgical mesh could be designed to modulate these key proinflammatory cytokines. In this way, while supporting the pelvic structure, the mesh could simultaneously function in controlling the cytokine response to minimize biomaterial rejection.

Table 1 describes the accuracy of supervised machine learning models and demonstrates that cytokines can exhibit predictive capabilities. Previous studies have performed predictive analysis for POP using risk factors derived from patient medical history [13]. However, such data can be incomplete and inaccurate [29]. This study demonstrates the utility that measured responses of biological samples can also have in developing robust predictive models. Chen et al [12] similarly used blood cytokine levels in a machine learning study to predict severe AKI after cardiac surgery. Their study concluded that a logistic regression model was the most effective in discovering the cytokine associations in severe AKI. In this study, the prediction accuracy for all 4 models exceeded 60%, with the artificial neural network model demonstrating the best overall performance, predicting POP surgical outcomes with 83% (15/18) accuracy when trained with all 13 cytokines. This predictive capability is similar to that reported for prediction derived from patient medical history [4], despite this study comprising a significantly smaller patient group. Considering the small population size, these results represent relatively high prediction accuracy for health care data.

When creating models trained with a subgroup of 7 cytokines (Table 2), selected using a random forest method, the artificial neural network model maintained the greatest effectiveness with respect to sensitivity, specificity, and prediction accuracy. Moreover, the group of selected cytokines outperformed the larger group of cytokines in the logistic regression model and achieved the same results in the decision tree model. The Naïve Bayes and artificial neural network prediction accuracy dropped only 5% when using the subgroup of cytokines, thus demonstrating the resiliency of these models. These results demonstrate that predictive capabilities are retained with fewer cytokines, which would enhance clinical feasibility by reducing the cost and time associated with this clinical decision tool.

### Limitations and Future Directions

This study implemented rigorous research methods to identify physiological relationships among cytokine markers and developed robust machine learning models to predict mesh exposure; yet, some limitations should be noted. First, because this is a pilot study, the sample size is limited to 20 participants within a single hospital system. Nevertheless, this limited sample size predicted 83% (15/18) accuracy, a level that compares favorably with another predictive model study by Chu et al [9] that achieved a prediction accuracy of 71% in a study population size of 467. Thus, the results of this pilot study indicate the utility of this approach and the merit of future studies. Future study will provide validation with a larger population of participants from multiple hospitals. Additionally, the 10 participants in each group were not matched regarding variables. To minimize confounders, patients with POP recurrence or taking medication that would alter inflammatory response were excluded and the age ranges and average age at the time of surgery within each group were similar. Future studies with a larger population, however, will benefit from matching participants with respect to these and other potentially confounding variables. Nonetheless, the results of this pilot study highlight the importance of inflammatory markers in the prediction of this postsurgical condition.

### Conclusions

While this preliminary study is limited to a sample size of just 20 participants, this novel approach to using cytokine response to predict POP surgical outcomes has successfully distinguished important cytokines and their correlations. Moreover, these relationships point toward prospective therapies that could promote better surgical outcomes. Supervised learning models also demonstrate a high level of accuracy, specificity, and sensitivity, even when a smaller group of cytokine data is used. This result suggests that blood cytokine analysis might be feasibly used in a clinical setting to predict POP surgical outcomes. Further study with a larger patient population will be needed to confirm the utility of this method. If successful at a larger scale, this approach has the potential to change perspectives in which surgeons would recommend and proceed with POP repair surgeries and to prevent undesired outcomes of mesh-related surgeries in patients.

## Abbreviations

AKI: acute kidney injury
GM-CSF: granulocyte-macrophage colony-stimulating factor
IFN: interferon
IL: interleukin
IRB: institutional review board
LPS: lipopolysaccharide
PC: principal component
PCA: principal component analysis
POP: pelvic organ prolapse
TNF: tumor necrosis factor

## Data Availability

Data are openly available in the GitHub public repository [30].
